# Addressing the contribution of small molecule-based biostimulants to the biofortification of maize in a water restriction scenario

**DOI:** 10.3389/fpls.2022.944066

**Published:** 2022-08-31

**Authors:** Alba E. Hernandiz, David Jiménez-Arias, Sarai Morales-Sierra, Andres A. Borges, Nuria De Diego

**Affiliations:** ^1^Laboratory of Plant Growth Regulators, Faculty of Science, Palacký University, Olomouc, Czechia; ^2^Centre of Region Haná for Biotechnological and Agricultural Research, Czech Advanced Technology and Research Institute, Palacký University, Olomouc, Czechia; ^3^ISOPlexis, Centro de Agricultura Sustentável e Tecnologia Alimentar, Campus Universitário da Penteada, Universidade da Madeira, Funchal, Portugal; ^4^Chemical Plant Defence Activators Group, Department of Life and Earth Science, IPNA-CSIC, Campus de Anchieta, San Cristóbal de La Laguna, Spain; ^5^Grupo de Biología Vegetal Aplicada, Departamento de Botánica, Ecología y Fisiología Vegetal-Facultad de Farmacia, Universidad de La Laguna, San Cristóbal de La Laguna, Spain

**Keywords:** drenching, mineral nutrition, polyamines, yield, *Zea mays*

## Abstract

Biostimulants have become an asset for agriculture since they are a greener alternative to traditionally used plant protection products. Also, they have gained the farmers’ acceptance due to their effect on enhancing the plant’s natural defense system against abiotic stresses. Besides commercially available complex products, small molecule-based biostimulants are useful for industry and research. Among them, polyamines (PAs) are well-studied natural compounds that can elicit numerous positive responses in drought-stressed plants. However, the studies are merely focused on the vegetative development of the plant. Therefore, we aimed to evaluate how drenching with putrescine (Put) and spermidine (Spd) modified the maize production and the yield quality parameters. First, a dosage optimization was performed, and then the best PA concentrations were applied by drenching the maize plants grown under well-watered (WW) conditions or water deficit (WD). Different mechanisms of action were observed for Put and Spd regarding maize production, including when both PAs similarly improved the water balance of the plants. The application of Put enhanced the quality and quantity of the yield under WW and Spd under WD. Regarding the nutritional quality of the grains, both PAs increased the carbohydrates content, whereas the contribution to the protein content changed by the interaction between compound and growth conditions. The mineral content of the grains was also greatly affected by the water condition and the PA application, with the most relevant results observed when Spd was applied, ending with flour richer in Zn, Cu, and Ca minerals that are considered important for human health. We showed that the exogenous PA application could be a highly efficient biofortification approach. Our findings open a new exciting use to be studied deep in the biostimulant research.

## Introduction

Plants are sessile organisms exposed to a rapidly changing environment. They respond to external stimuli, which might result in plant acclimation to specific growing conditions. When this is impossible, growth becomes inhibited and, later, may die. Abiotic stresses are the principal cause of severe yield losses of 50–80%, depending on the crop and geographical location (Zhang et al., 2018). The global climate change projections forecast an increase in extreme weather events’ occurrence, frequency, and severity (FAO, 2018). The incidence of abiotic stresses such as drought will raise and compromise the yield of the crops, especially in arid and semiarid areas. Drought is multidimensional stress affecting plants at various developmental stages, including the plant’s production (Blum, 1996). One of the most promising methods to cope with the inevitable abiotic stresses is the application of biostimulants to enhance plant resilience to environmental perturbations (Van Oosten et al., 2017). Their action relies on the “preparation” effect (priming or hardening) that their application exerts on the plants (Gebremedhn and Berhanu, 2013; Maiti and Pramanik, 2013; Savvides et al., 2016). Biostimulants have been proved to improve plant growth and photosynthesis efficiency by modifying the plant metabolism under abiotic stress conditions (Paul et al., 2019; Sorrentino et al., 2021). Moreover, the recent recognition of biostimulants as an independent group of agricultural inputs by the European Union and their contribution to more sustainable agricultural practices forecasts a growing interest in these substances (Ben Mrid et al., 2021).

Typically, biostimulants are a mixture of several substances, such as protein hydrolysates or seaweed extracts; this has been seen as a great opportunity by some companies to join the circular economy trend since they can give a second life to waste and by-products (Xu and Geelen, 2018). However, these products present the disadvantage of lack of uniformity between the batches and the problematic identification of the active substances (Yakhin et al., 2017). Studying pure organic active compounds as biostimulants will lead to a better standardization, the quality control of formulation, and an understanding of their mode and mechanism of action (García-García et al., 2020). While the effectiveness of biostimulants has been widely researched and summarized in extensive reviews (Battacharyya et al., 2015; Bulgari et al., 2015, 2019), there is a limited information available on how their application might affect the final yield and quality.

Maize (*Zea mays* L.) is one of the most important cereal crops for human nutrition in large parts of the world (Huma et al., 2019) and an essential grain used as livestock feed in the world (Loy and Lundy, 2018). Drought negatively affects the plant growth, the dry biomass content, and the yield (Anjum et al., 2017; Kim et al., 2019). The water deficit during maturation might cause problems in the grain filling stage (Zhang et al., 2019) and reduce the quality parameters such as starch, proteins (Barutcular et al., 2016), or mineral content (Aqaei et al., 2020). Maize kernel mainly comprises carbohydrates, protein, and oil (Chaudhary et al., 2014). Starch accounts for approximately 70% of the kernel weight (Orhun et al., 2013). According to the release and absorption of glucose in the intestines, starch is divided into the following three categories: Rapidly and slowly digestible starch and resistant starch; the last category has particular importance for human nutrition since its consumption has been linked to a decrease in the risk of developing type 2 diabetes and colon cancer (Hoebler et al., 1999). Protein content varies from 8 to 11% of the kernel weight on common varieties of maize (Landry and Moureaux, 1980). However, the nutritional value is poor for monogastric and human consumption due to the low content of essential amino acids such as lysine, tryptophan, and threonine (Li and Vasal, 2015). The discovery of the opaque-2 gene and its link with higher lysine and tryptophan was the beginning of the Quality Protein Maize (QPM) research, which produced hybrids whose kernels had a significant increment on the mentioned amino acids. Nevertheless, the yield was reduced in these hybrids, and their agronomical performance was deficient (Chaudhary et al., 2014). The kernel mineral composition is strongly affected by the environmental factors, soil moisture, and fertility, but it is mainly genotype dependent, with the vast variety of differences as the most significant contributor to the reported variance (Feil et al., 2005; Menkir, 2008).

The development of nutritionally enhanced food crops using traditional breeding practices and modern biotechnology approaches is known as “biofortification” (Chaudhary et al., 2014; Garg et al., 2018). The first main achievements in this field were the lysine and tryptophan enriched maizes or vitamin A-rich orange sweet potato; much effort is put into transgenic research, although traditional breeding practices are best accepted (Garg et al., 2018). Given the exposed facts, we decided to test biostimulants as an alternative approach for maize biofortification due to their effect on plant metabolism and stress tolerance. With this aim, we applied two polyamines (PAs) with proven efficiency as stress alleviators, putrescine (Put), and spermidine (Spd) (Li et al., 2015, 2018; Marcińska et al., 2020; Islam et al., 2022), although the dose was optimized to the specific cultivar of maize used. The maize plants were treated with the two PAs *via* drenching, which has been proven to have positive results toward drought stress (Jiménez-Arias et al., 2019) because drenching provides a more extended period of continuous uptake of the supplemented substance than the foliar application (Parkunan et al., 2011). Some reports also revealed that the drench application improves soil fertility by increasing the availability of minerals, soil aeration, and water holding capacity and promotes the development of essential microbes (Battacharyya et al., 2015; du Jardin, 2015). We expect that applying PAs by drenching will improve maize’s biofortification and stress tolerance.

## Materials and methods

### Plant material, growth conditions, and dose optimization

A local plant nursery provided a local forage variety of maize from Gran Canaria Island (*Zea mays* L. c.v. Lechucilla) in a 150-socket nursery tray. One week after sowing, the plants were placed in a growth chamber under controlled conditions with a temperature of 22°C, a photoperiod of 16-h light and 8-h dark, with a light intensity of 300–400 μmol m^–2^ s^–1^, and a relative humidity around 60–70%. Plants in stage V1 were used for the dose optimization experiments when the lowermost leaf had a visible leaf collar (Zhao et al., 2012).

Putrescine and spermidine were purchased from Sigma–Aldrich (Put CAS number 333-93-7; Spd CAS number 124-20-9). In the direct application-optimized doses of both chemicals under two water regimes in the nursery tray, 20 plants were used as biological replicates per variant (treatment × growth condition), as described in Jiménez-Arias et al. (2022). The nursery experiment ensured the suitability of the plant for the root treatment. The treatments were applied directly to the roots of stage V1 plants, consisting of 5 ml of a half-strength Hoagland solution (Hoagland and Arnon, 1938) for the controls or containing the tested substances at the following five concentrations: 0.01, 0.1, 0.5, 1, and 2 mM for the treated plants. Twenty plants per variant were grown under a water limitation of 50% of the field capacity for a week. The two harvesting times were performed; at the dry period’s beginning and end. The whole plant, including the aerial part and roots, was harvested. The dried weight (DW, mg) of the 20 plants was recorded after being oven-dried at 85°C for 48 h to calculate the relative growth ratio (RGR) (Hoffmann and Poorter, 2002),


(1) $$\text{RGR}=\left(\ln\,{\mathrm{DW}}_{2}-\ln\,{\mathrm{DW}}_{1}\right)/\left(t_{2}-t_{1}\right),$$

where DW_1_ and DW_2_ corresponded to the dry weights of maize seedlings at times *t*_1_ and *t*_2_ (beginning and end of the water deficit).

The plant water use efficiency (WUE, mg ml^–1^) was calculated considering all the amount of water provided during the experiment timespan (Kuglitsch et al., 2008).


(2) WUE=Plant⁢biomass/Total⁢irrigation 

### Greenhouse experiment

Once the data was evaluated for the dosage optimization, an additional study with maize variety was conducted at a greenhouse property of the Escuela de Capacitación Agraria de Tacoronte (Tenerife), Canary Islands (28°29′47.0′′N 16°25′12.0′′ W) from June 2021 to August 2021. The ambient conditions were recorded daily (Supplementary Table 1). The average maximum and minimum temperatures were 30 and 22°C, respectively, with an average relative humidity of 80% (Supplementary Table 1). The soil is classified as clay–loam (35% clay, 27% silt, and 38% sand), and the experiment was organized in randomly distributed blocks of 20 m^2^ blocks with three replicates, each block containing 80 plants. The seeds were sown in nursery trays and transplanted to the field after 15 days of the sowing. The irrigation was calculated according to the FAO (Rao et al., 2016), taking into account the evapotranspiration rate (ETo) provided by a nearby meteorological station, property of the island council, Cabildo de Tenerife (Supplementary Table 1). The soil humidity was monitored within the wet-bulb with the TEROS 12 FDR sensor (METER Group, Pullman, WA, United States). The treatments consisted of 20 ml of water for the controls or of 0.1-mM Put (CAS No.: 333-93-7) or 0.5-mM Spd (CAS No.: 124-20-9) for the treated plants; both compounds purchased from Aldrich Chemical Co. (St. Louis, MO, United States). The treatment was applied manually using a backpack sprayer with a dispenser. The solutions were applied directly to the wet soil, between the irrigation emitter and the plants, to ensure the direct availability of the root system. The treatments were applied twice, 2 and 4 weeks after the transplanting. After the second application, the water restriction for the drought variant started, and these conditions were maintained until the harvest of the maize cobs. The plants did not receive additional fertilization or protective treatment during the experiment. The weeds were manually removed every 7–10 days to avoid competition for the water.

#### Biomass, yield, and water status measurement

In 15 and 30 days after the onset of the water restriction, the relative water content (RWC, %) was calculated (Barrs and Weatherley, 1962). A total of 20 disks of 1-cm diameter per variant were excised from the last fully expanded leaf and immediately weighted to register the fresh weight (FW, mg). After that, the disks were submerged in distilled water for 24 h to determine the turgid weight (TW, mg). Finally, the disks were oven-dried at 85°C for 48 h to register the dry weight (DW, mg). The RWC for each disk was calculated as


(3) RWC=(FW-DW)/(TW-DW) 

The yield-related parameters were evaluated at the harvest, 45 days after the onset of the stress. The number of adequately developed maize cobs per plant and variant was counted. Ten random plants per variant were selected to determine morphometric parameters such as plant length (from insertion to the soil until the base of the flower) and width (at the middle of the stem length), and the length and width of the last fully developed leaf. The cobs to evaluate production were also obtained from the selected plants. The cobs were weighed to record the fresh weight and then oven-dried at 65°C for 1 week to reduce the possible bias due to the different moisture levels. The dry cob weight, the number and total weight of all kernels per cob, and the weight of 100 kernels (in triplicate from each cob) were registered. The total yield considering 40,000 plants ha^–1^ for the used plantation frame was also calculated using the following equation with the following formula:


(4) $$\mathrm{Yield}\,{\mathrm{ha}}^{-1}=\mathrm{Average}\,\mathrm{kernel}\,\mathrm{weight}/\text{Cob×}\mathrm{Average}\,\mathrm{cob}\,\mathrm{number}/\mathrm{Plant}\times \mathrm{Estimated}\,\mathrm{plants}\,{\mathrm{ha}}^{-1}$$

The Harvest index (HI) was also calculated from the total kernel weight (total KW) per cob and the average cob fresh weight (average cob FW) as follows:


(5) HI=Total⁢KW/Average⁢Cob⁢FW

The production water use efficiency (WUEp) in maize was also estimated (Kiziloglu et al., 2009). The accumulated effective crop evapotranspiration (ETc, mm) was calculated through the experiment (Supplementary Table 1). This term corrects the deficiencies of the ETo values by a Kc factor that depends on the moisture soil level, crop characteristics, and the stage of the crop vegetative cycle (6). For maize, the Kc values are estimated at 1.2 in the initial phases to 0.6 at the final stages (FAO).


(6) ETc=ETo⁢X⁢Kc

The WUEp (kg mm^–1^) was then calculated as the ratio between the accumulated ETc and the final yield (kg ha^–1^).

#### Protein, carbohydrates, and mineral composition of the maize flour

All kernels from the selected cobs were ground to a fine powder to evaluate quality parameters. A 50 mg of the powder per sample was used for the total protein content calculated using the Kjeldahl Method (Kirk, 1950), multiplying the total nitrogen content by 6.25. An additional 100 mg of the powder was used to determine total carbohydrates using the phenol sulfuric acid method modified in multi-well plates (Jiménez-Arias et al., 2019). Finally, 1 g of the flour powder per sample was used to analyze the mineral content (Ca, Mg, K, P, Na, Cu, Zn, and Fe). Each sample was converted to ash in a muffle stove at 480°C and mineralized by the dry method with 6 N HCl. The mineral levels were determined by ICP OES Avio 500 (Perkin Elmer, Waltham, MA, United States).

### Data analysis

All plant growth and production parameters were used to calculate the Plant Biostimulant Characterization Index (PBCI), a visual and helpful tool to reduce all considered variants into a single number a for better biostimulant characterization (Ugena et al., 2018; Sorrentino et al., 2021). The PBCI was used to select the best-performed concentrations in the first experiment (dose optimization) and evaluate the PA application impact on maize production.

The data were evaluated using different statistical approaches. First, using Infostat software, the normality of the variables was assessed with the Shapiro–Wilks test. After that, a two-way ANOVA (*p* ≤ 0.05) followed by multiple comparisons with LSD posthoc test was used for parametric data and Kruskal–Wallis test (α = 0.05) for nonparametric data. The multivariate statistical analyses with the yield-related parameters were also carried out for better visualization. One principal component (PC-Dim) analysis and matrix correlation were constructed in RStudio v.2021.09.1+372 using the packages *factoextra*, *ggplot2*, and *corrplot*.

## Results

### Nursery tray dose optimization

The dose optimization was performed in nursery trays following the protocol established by Jiménez-Arias et al. (2022). The parameters considered to evaluate the efficiency of the dose were the plant weight (shoot and root), the RGR (%), and WUE (Supplementary Table 2). Table 1 displays the heatmap from the computed parameters, transformed with the log2 and relativized to the untreated maize seedlings. The drenching with Put at 0.5 and especially 0.1 mM enhanced the plant biomass (weight), RGR (%), and WUE compared to untreated seedlings when grown under a water limitation of 50% field capacity, ending with a positive PBCI value (Table 1). Contrarily, the application of Spd did not improve the seedlings performance under the same growth conditions (Table 1 and Supplementary Table 2). The plants treated with 0.5-mM Spm presented less negative PBCI (Table 1). Thus, we selected 0.1-mM Put and 0.5-mM Spd to evaluate their impact on maize production under optimal and water deficit conditions.

**TABLE 1:** Heatmap summarizes the parameters estimated in maize seedlings treated with Put or Spd at five concentrations (0.01, 0.1, 0.5, and 1 or 2 mM) grown under water limitation (50% field capacity).

Compound	Concentration	Biomass	RGR	WUE	PCBI
Put	0.01	−0.003	−0.001	−0.003	**−0.008**
	0.1	0.050	0.026	0.059	**0.135**
	0.5	0.014	0.008	0.017	**0.039**
	1	−0.033	−0.018	−0.039	**−0.091**
	2	−0.021	−0.011	−0.025	**−0.057**
Spd	0.01	−0.089	−0.049	−0.106	**−0.244**
	0.1	−0.085	−0.046	−0.101	**−0.232**
	0.5	−0.044	−0.024	−0.052	**−0.120**
	2	−0.078	−0.042	−0.092	**−0.212**
	1	−0.119	−0.066	−0.143	**−0.328**

The studied parameters were plant dried weight (Plant W, mg), relative growth ratio (RGR, %), and water use efficiency (WUE, mg mL^–1^). The table represents the ratio (log2) between the original values of each variant and the untreated plants (Control). The right panel shows the obtained PBCI values. Blue indicates a stress alleviator; orange indicates a stress inductor.

### The application of polyamines enhanced the RWC in maize plants

**FIGURE 1:**
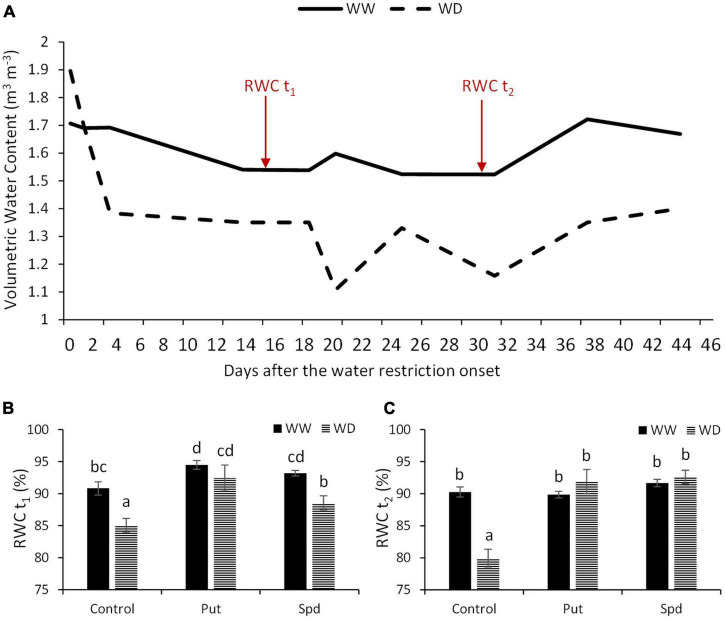
Water-related parameters in maize plants under optimal conditions and water limitation. **(A)** Volumetric water content of the soil (m^3^ m^–3^) measured with the FDR soil moisture sensor from the onset of the water restriction. Red arrows indicate the two dates (*t*_1_, *t*_2_), when the relative water content (RWC, %) was measured in the maize plants untreated (Control) and treated with 0.1-mM Put or 0.5-mM Spd under optimal conditions (WW) or water deficit (WD). **(B)** RWC*t*_1_ of the plants, *t*_1_ corresponds to15 days after the onset of the stress). **(C)** RWC*t*_2_ (%) of the plants, *t*_2_ corresponds to 30 days after the onset of the stress. Mean ± SE; *N* = 24. Different letters for the RWC values indicate the significant differences according to the LSD test after two-way ANOVA (*p* ≤ 0.05).

The changes in soil water content were recorded through the experiment and represented in Figure 1A. The plants under well-watered conditions were irrigated according to their water requirements estimated by FAO’s reference evapotranspiration (ETo) (Supplementary Table 1). The water supply was reduced by 20% of the crops water requirements for the water stress variants. As a result, we observed that the volumetric water content in the well-watered plants was maintained between 1.7 and 1.6 m^3^ m^–3^ (Figure 1A). However, in the stress variant, the water was drastically reduced to a volumetric water content of 1.4 m^3^ m^–3^ for the first four days and maintained for additional 15 days. After that, it fluctuated between 1.3 and 1.1 m^3^ m^–3^.

The water status of the plants was estimated twice by the RWC, at 15 (t_1_) and 30 (t_2_) days after the water restriction onset (Figures 1B,C). According to ANOVA, there was a significant interaction between growth conditions and treatment for RWCt_2_ but not for RWCt_1_. The water restriction reduced the RWCt_1_ in all plants compared to well-watered ones. However, the application of Put and Spd increased the RWCt_1_ values compared to their respective controls. The Put and Spd applications kept the same trend, with an RWCt_1_ of 4 and 2.6% for WW and 9 and 4% for WD conditions, respectively, compared to the corresponding untreated plants (Supplementary Table 3). Interestingly, no differences in RWCt_2_ were observed among variants; only the untreated plants significantly reduced the RWCt_2_ to 80% (Figure 1C and Supplementary Table 3). Thus, Put and Spd applications increased significantly RWCt_2_ to 14.9 and 15.8% in the maize plants, respectively, compared to the untreated plants under WD (Supplementary Tables 1, 3).

### The application of Spd by drenching reduced the biomass-related parameters under water limitation

**FIGURE 2:**
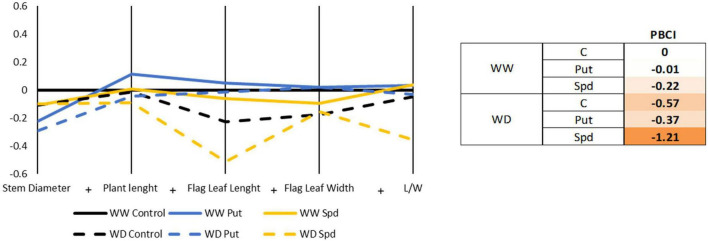
Parallel coordinate plot and PBCI regarding the biomass-related parameters. Stem diameter (mm), plant length (cm), flag leaf length and width (cm), and length/width ratio (L/W) of maize plants treated with 0.1-mM Put or 0.5-mM Spd under optimal conditions (WW) or water deficit (WD). The plot represents the ratio (log2) between the original values of each variant and the untreated plants under WW conditions. The right panel shows the obtained PBCI values. Orange color indicates stress inductor.

The biomass production was evaluated by monitoring several parameters such as stem diameter, the plant length (measured from the transition to the roots, up to the emission of the anthers), length and width of the flag leaf, and the ratio length/width (L/W) for the flag leaf (Figure 2 and Supplementary Table 4). The parameters were then represented in a parallel coordinate plot, and the values were used for the PBCI calculation. None of the treatments enhanced plant biomass production in any water regime, so all presented negative PBCI values (Figure 2). The parallel coordinate plot illustrated that only Put slightly enhanced the biomass production under WW conditions; the rest of the treatments and growth conditions presented a negative effect. The thicker stem was reported for the WW control plants, with Put plants having the thinnest stem for both irrigation conditions. Interestingly, the Spd-treated plants were the only ones keeping the stem thickness irrespectively of the growth conditions (Figure 2 and Supplementary Table 4). The water limitation slightly reduced the plant height, flag leaf length, and width without significant differences (Supplementary Table 4). Overall, the highest plant length and leaves were found in the Put treated maizes under WW conditions, while the shortest plants were reported for the WD with Spd application. Regarding the leaf L/W ratio, the highest values were reported for Put and Spd in WW conditions, followed closely by the Control treatment and Put under WD (Figure 2 and Supplementary Table 4). The lowest L/W ratio was observed in the maize plants treated with Spd under WD, confirming that this treatment modified the plant stress response and hence, the growth profile.

### Put enhanced yield and WUEp under optimal conditions, and Spd under water limitations

**FIGURE 3:**
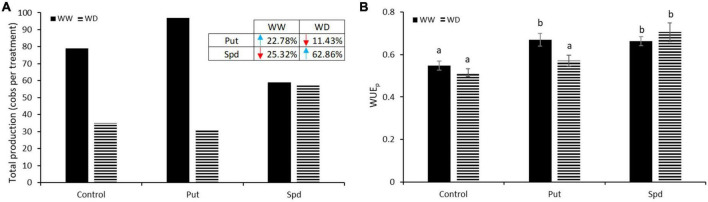
Total production and WUEp in PA treated maize plants. **(A)** Total production expressed as the sum of all cobs produced per variant in maize plants untreated (Control) and treated with 0.1-mM Put or 0.5-mM Spd grown under well water (WW) or water deficit (WD). Enclosed in the left upper table, the percentage of increment (blue, upside arrow) or reduction (orange, downside arrow) of Put and Spd treatment relative to the respective control in WW and WD conditions. **(B)** Water use efficiency calculated as the ratio between the fresh biomass production and the ETo adapted to maize crop with the FAO Kc. Different letters indicate the significant differences between the treatments and the growth conditions according to the LSD test after two-way ANOVA; *p* < 0.05.

As a first step in evaluating maize’s yield, the total number of cobs produced per variant was counted and represented in Figure 3A. The Put application was the most effective treatment under WW, so its plants increased the total production by 22.78%, with 97 cobs compared to 79 obtained in the controls. On the other hand, Spd reduced the production to only 59 cobs. Water limitation reduced the final yield in all treatments, except in Spd treated plants that maintained similar production as observed under WW conditions (Figure 3A) and enhanced the production by 62.78% compared to control plants under WD. However, the Put application reduced plant production by 11.43% (Figure 3A).

Crop physiologists initially considered WUE as the amount of carbon assimilated and crop yield per unit of transpiration (Viets, 1962), although the definition evolved to biomass or marketable yield produced per unit of transpiration. In this sense, this parameter is essential to better characterize the productivity of crops, especially in a water scarcity scenario. In this work, the production WUE was calculated to understand better the relationship between plant production and the water used (Figure 3B). A significant interaction between treatment and growth condition was obtained according to ANOVA (*p* = 0.002). In control plants, no differences in WUEp were observed among growth conditions. However, the PA treatments enhanced the WUEp of the treated plants under WW conditions, but only Spd application improved this parameter under WD (Figure 3B). These results pointed to WUEp increase induced by PAs as one of the primary factors conditioning maize yield under optimal and water limitation conditions.

**FIGURE 4:**
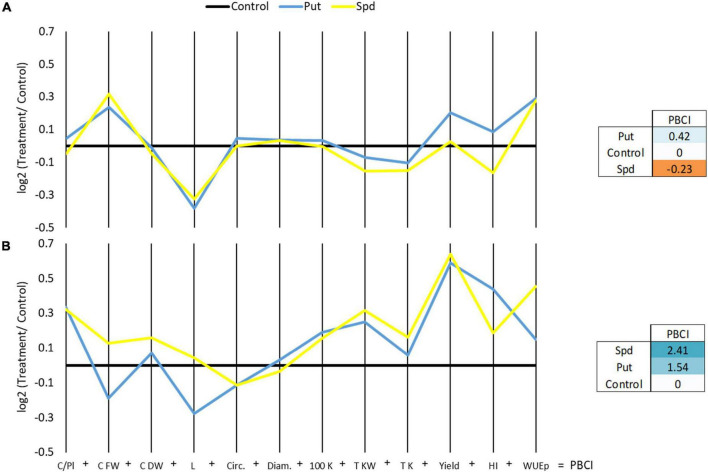
Parallel coordinates plot representing log2 of the production parameters relativized with the control of each growth condition; cobs per plant (Cobs/Pl), cobs fresh (Cob FW), and dry weight (Cob DW) (g), length [L(K)] (kernels in a line), circumference [circ. (K)] (kernel circumference) and diameter (diam.) (mm), the weight of 100 kernels (100 K DW) (g) and the total kernel weight (Total KW) (g), the total number of kernels (Total K), the yield per hectare, considering the dry kernel production [Yield(K) ha^–1^], harvest index (HI) (Total dry kernels weight/Average cob fresh weight), and the WUE in maize plants untreated (Control) and treated with 0.1-mM Put or 0.5-mM Spd grown under well water (WW) **(A)** or water deficit (WD) **(B)**. The right panel represents the PBCI, which summarizes the plot in a single number positive (Blue color) for growth promotor or negative (orange) for stressor.

Other parameters related to the cobs and kernel production were also determined (Supplementary Table 5). Among them, the fresh weight and length of the cobs and the final yield per hectare considering the kernel were the production-related parameters that showed a significant interactive effect on the treatment and growth conditions according to ANOVA (*p* ≤ 0.05). The treatment with Put and Spd improved the fresh cob weight but reduced the length compared to the controls under WW, but not under WD (Figure 4 and Supplementary Table 5). Contrarily, the PA treatments reduced the cob length in both growth conditions except for Spd under WD. Polyamines significantly increased the yield per hectare (1.5 × 10^6^ g ha^–1^ for Put and 1.6 × 10^6^ g ha^–1^ for Spd) compared to untreated plants (1 × 10^6^ g ha^–1^) under WD conditions, reaching the level of the WW plants (Figure 4 and Supplementary Table 5). Regarding the harvest index (HI), Put-treated plants under WD overcame the levels of controls under WW conditions.

To integrate the production data, two additional PBCIs were estimated (right panel, Figure 4). As a final result, we observed that Put improved maize yield (positive values for PBCI) under WW and WD conditions. However, Spd only enhanced maize production under WD conditions and negatively affected the plants under WW (right panel, Figure 4). Altogether, the PA application by drenching enhances the quality of maize production, especially under water restrictions.

### Polyamines application modifies the quality of the maize flour

**FIGURE 5:**
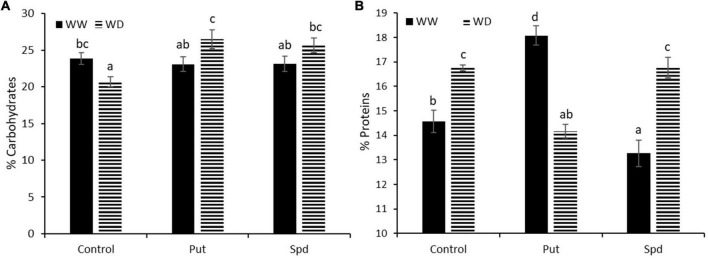
Content of carbohydrates **(A)** and protein **(B)** expressed as % of the weight of the sample WUE in maize plants untreated (Control) and treated with 0.1-mM Put or 0.5-mM Spd grown under well water (WW) or water deficit (WD). Different letters indicate the significant differences between the treatments and growth conditions according to the LSD test after two-way ANOVA, *p* < 0.05.

As the final step, we evaluated the percentage of carbohydrates (CH) content and protein in the kernel powder obtained from each treatment and growth conditions (Figure 5 and Supplementary Table 6). According to ANOVA, both parameters were affected by the interaction between the treatment and growth conditions. Under WW conditions, the PA application did not change the carbohydrate content (Figure 5A). The reduced water availability significantly decreased the CH content for the control treatment; curiously, both PA treatments resulted in significantly higher CH content than the WD control, leveling it up to the control treatment under WW conditions (Supplementary Table 6). The water availability significantly affected the protein content (%), with higher values for the kernels from the controls under WD than under WW conditions (Figure 5B). The PA application presented a different pattern (Supplementary Table 6). Also, Put significantly increased the protein content under WW conditions but reduced them under WD compared to the respective controls. Contrarily, Spd did not affect the protein content under WD but significantly reduced it under the protein content compared to the controls under WW conditions (Figure 5B).

The mineralogical profile of the flour obtained from the dry grains was also analyzed (Table 2 and Supplementary Table 7). Significant changes were observed in the flour mineral compositions due to the treatment, growth conditions, and their interaction. As Na, P, and Cu were the most sensitive parameters because their changes were due to the interaction between the treatment and the growth conditions (*p* ≤ 0.006, *p* ≤ 0.001, and *p* ≤ 0.047, respectively). The treated plants presented the biggest changes in Cu, mainly due to a significant increment in those treated with Spd (Table 2 and Supplementary Table 7). The WD conditions significantly reduced the Na levels of the non-treated plant, but the application with Put and Spd kept them at the same levels as the WW-variants. The water restriction significantly induced the P, K, and Mg accumulation in the plants under WD, except the Spd treated ones that kept the lower values observed in the flour from the WW plants. The opposite situation was observed for Ca, where only the Spd-treated plants from WD kept the levels of the WW ones (Table 2 and Supplementary Table 7). Altogether, the PA application modifies maize flour’s chemical and mineral composition. However, the changes are also highly influenced by the growth conditions.

**TABLE 2:** Heatmap containing the log2 values of the different mineral elements content (ppm) in maize plants untreated (Control) and treated with 0.1-mM Put or 0.5-mM Spd grown under optimal conditions (WW) or water deficit (WD).

Growth conditions	Treatment	Ca	Fe	K	Mg	Na	P	Zn	Cu
WW	Control	0 ab	0 a	0 ab	0 ab	0 b	0 ab	0 a	0 a
	Put	0.58*ab*	−2.00a	−0.42a	−0.24a	0.00b	−0.15a	−1.77a	0.77a
	Spd	0.20*ab*	0.38a	−0.27*ab*	−0.11a	−0.07b	0.14*bc*	1.45b	0.31a
WD	Control	−0.42a	−0.70a	0.33c	0.25c	−0.22a	0.24c	−0.41a	1.23a
	Put	−0.14*ab*	0.17a	0.14*bc*	0.22*bc*	0.03b	0.23*bc*	−0.48a	0.19a
	Spd	0.72b	−1.86a	−0.17*ab*	−0.15a	−0.03b	−0.08a	−1.53a	2.46b

The values are relative to the control treatment under WW. The different letters indicate significant differences according to the LSD test after two-way ANOVA, p = 0.05. Blue indicates accumulation and red reduction.

### The multivariate statistical analysis uncovers the different effects of Put and Spd in maize

**FIGURE 6:**
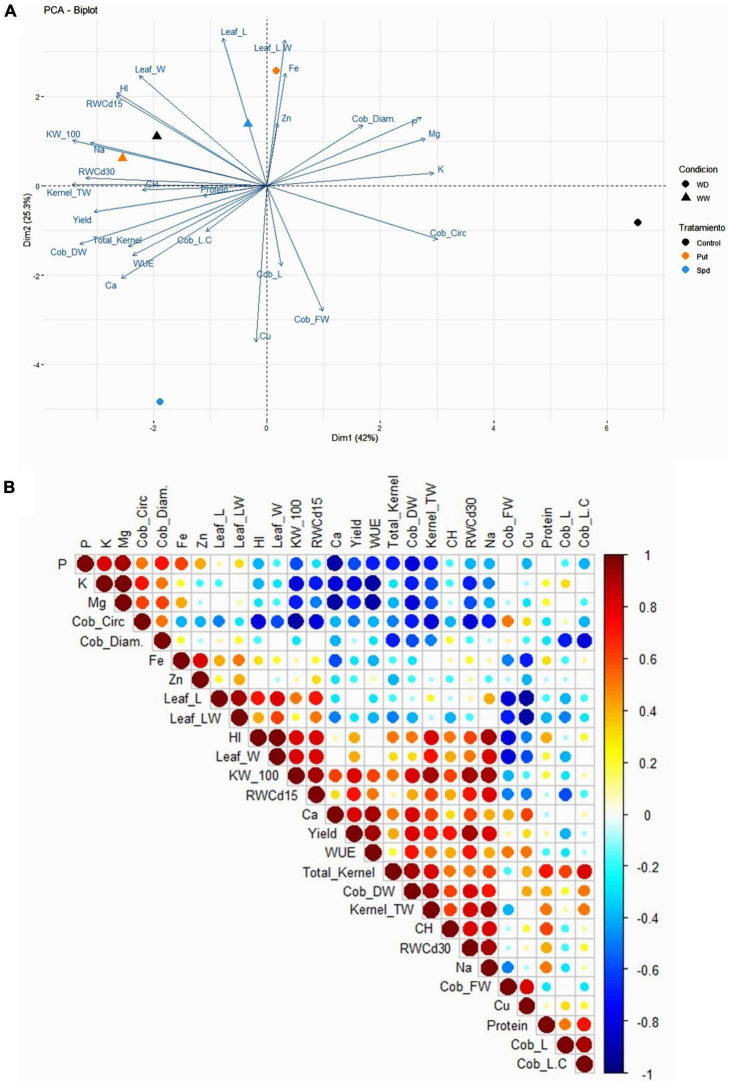
Principal component analysis **(A)** and correlation matrix **(B)** of the studied biomass parameters [flag leaf length (Leaf_L), flag leaf width (Leaf_W), and flag leaf length/width ratio (leaf_LW)], relative water content at 15 and 30 days after the stress onset (RWC 15d and RWC30d), productivity parameters [cob length (Cob_L), cob circumference (Cob_circ.), cob diameter (Cob_diam.), cob fresh weight (Cob_FW), cob dry weight (Cob_DW), weight of 100 kernels (KW_100), total number of kernels per cob (Total_Kernel); total weight of the kernels per cob (Kernel_TW), harvest index (HI), yield, water use efficiency (WUE), protein content (Protein), carbohydrates content (CH), and mineralogical profile of the kernels (P, K, Mg, Fe, Zn, Ca, Na, and Cu) in maize plants untreated (Control) and treated with 0.1-mM Put or 0.5-mM Spd grown under well water (WW) or water deficit (WD).

To better visualize and integrate the biomass, productivity, hydric status of the plants, and the kernel nutritional profile, we performed a principal component analysis (PCA) and correlation matrix (Figure 6). The two first PCs explained 67.3% (Dim1 = 42%; Dim2 = 25.3%) of the total model variation. As the first result, Dim1 separated the non-treated plants due to the irrigation regime (Figure 6A). However, whereas stressed Put treated plants were located close to the irrigated plants, Spd was located opposite to these plants, pointing to a different mechanism of action between these two PAs. Put treated plants under WD strongly correlated with the leaf biomass expressed as flag leaf length/width ratio and the Zn and Fe levels. This result was also evident in the negative correlation between Fe, p, K, and Mg with many production-related parameters, the plant RWC and Ca in the flour, observed in the correlation matrix (Figure 6A). Contrarily, Spd-treated plants under WD presented longer (Cob_L) and heavier (Cobs_FW) cobs, with higher content of Cu in the flour (Figure 6A). It is worth mentioning that the growth condition affected CH and protein content, presenting a higher correlation with the RWC, the kernel-related parameters, and the final yield. The PA-activated strategy conditioned the biomass production (vegetative or reproductive biomass) and the composition of the final product; in this case, the quality of the flour.

## Discussion

Nowadays, the research topics need to focus on understanding how the principal abiotic stresses such as drought affect crop yield. Besides, due to the promising biostimulant efficiency to mitigate the plants’ stress effect, new evidence is needed to know if they provide any quantitative or qualitative benefit in the final yield. This study aimed to evaluate using the major PAs as small molecules-based biostimulants to improve maize production under optimal and water restriction conditions. Their impact on maize biofortification is also investigated. A recent review has reported the relevance of these compounds in regulating plant tolerance/resistance to stress abiotic/biotic (Ramazan et al., 2022). When used PAs as a foliar application, the concentration range of 0.1–1 mM elicited positive responses in stressed plants (De Diego and Spíchal, 2020). In this work, we investigated their application *via* fertirrigation because it is considered an efficient agricultural method to enable plants to cope with the consequences of the water limitation during the growth and fruit production (Agliassa et al., 2021). The main reason is that the fertirrigation provides the plant with a longer nutrient uptake window than foliar application (Parkunan et al., 2011). However, it must be considered that different application methods can trigger different responses in the plants (Paul et al., 2019), so optimizing the dose for the root treatment was an essential step, ending with 0.1-mM Put and 0.5-mM Spd the most effective concentrations (Table 1).

The application of PAs reduced the RWC losses when plants are subjected to water limitations (Figure 2) compared to untreated plants. Many studies have also obtained similar results and reported that the PA-induced better water balance is due to decreasing the stomatal conductance and increasing proline, anthocyanins, and soluble phenolics levels, improving membrane properties and enhancing the activity of catalase and superoxide dismutase (Farooq et al., 2009; Hassan et al., 2018). Their application has also been described as improving plant osmotic adjustment mechanisms (Choudhary et al., 2022). However, the improvement in the water balance did not influence the plant biomass but instead reduced it in the PA-treated plants (Figure 3). Similar results were also observed in other maize species (Li et al., 2018) and crops (Ullah et al., 2012; Liu et al., 2018). One possible explanation is that this type of treatment simulates moderate stress in the plants, so-called hardening, to be ready for fighting future adverse conditions (Duarte-Sierra et al., 2020).

**FIGURE 7:**
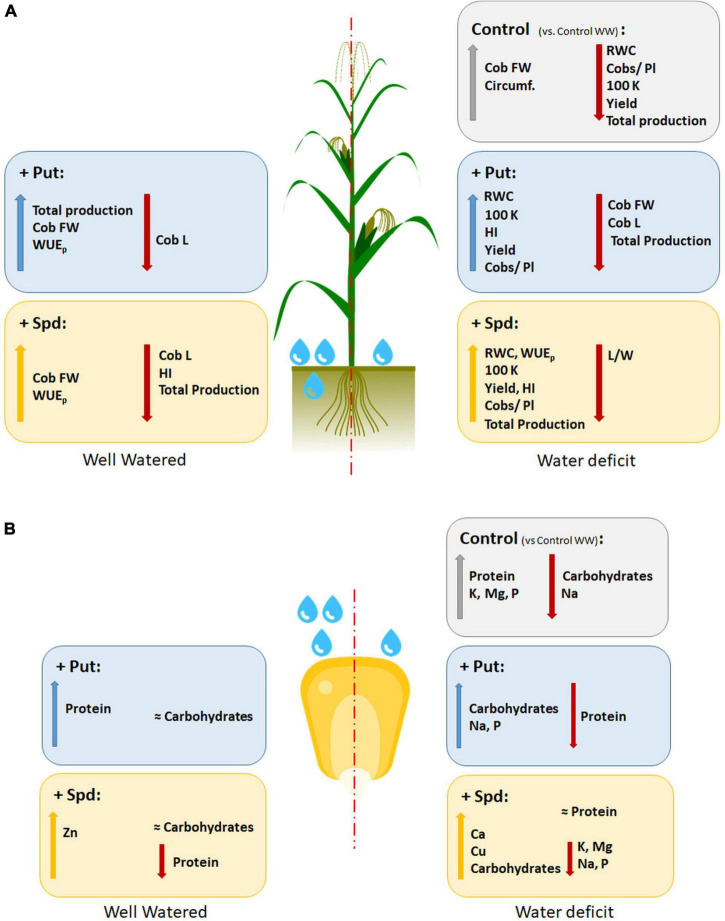
Summary of the variation of the observed parameters in maize plants untreated (Control) and treated with 0.1-mM Put or 0.5-mM Spd grown under well-water (WW) or water deficit (WD). Bold formatting of the font means the statistical effect on the increment or reduction; the total production is expressed as the sum of every cob produced per treatment and condition. The parameters displayed are the water use efficiency (WUE); leaf relative water content (RWC); flag leave length/width ration (L/W); cob fresh weight (g) (Cob FW); cob length, as kernels in the longitudinal line (Cob L); cob circumference, as the kernels in the perimeter at the center of the cob (circumf.); average cobs produced in a plant (Cobs/pl); the weight of 100 kernels (g) (100 K); harvest index (HI); yield (10^6^g ha^–1^) (Yield); % of carbohydrates; % of protein; and minerals (ppm) such as Ca, Zn, K, Mg, Na, P, and Cu.

The “no improvement” of the plant growth might also be an energy-saving mechanism to redirect the PA-induced/accumulated resources to the enhancement of the production of the crop. This concept has already been proposed for fruit tree management, where the application of plant growth retardants has been explored to reduce vegetative growth and obtain higher production (Köhne, 1989). According to this assumption, we expected that both PA treatments improved maize production under WW and WD. However, different responses were observed; Put enhanced the total maize yield under WW but reduced it under WD (Figure 3). Despite the reduced total maize yield, drenching with 0.1-mM Put ended with a positive PBCI under WD due to a higher number of cobs per plant, higher kernel weight, or yield per ha (Figures 4, 7A and Supplementary Table 5). The exogenous application of PAs, including Put, has improved flowering and yield in many plant species (Reviewed by González-Hernández et al., 2022) and wheat production (Mostafa et al., 2010). These results could partially explain the higher number of cobs per plant. Besides, the exogenous application of 0.1-mM Put enhanced yield in winter wheat under WD and the plant biomass (Gupta and Gupta, 2011; Gupta et al., 2012). Besides, the long-term application of Put on salt-stressed rice stimulated the morphogenesis of reproductive structures, enhancing the yield compared to the unstressed plants (Ndayiragije and Lutts, 2007). This positive effect of Put was also observed in the vegetative development and yield of barley salt-stressed plants (Seleem et al., 2021). Pál et al. (2018) also suggested that Put pretreatment induces acclimation processes under controlled conditions. These results agree with ours, so the Put-treated plants presented the best growth and yield under control conditions, and they had higher biomass production and some productive parameters under WD (Figures 4, 6A, 7A).

Overall, the PA addition enhanced the production, in agreement with the findings of Liang and Lur (2002), who demonstrated that aborted maize kernels had lower endogenous PA levels. However, a much higher yield was obtained with Spd than with Put under WD (Figures 3, 4 and Supplementary Table 5). This could be because the vital biological processes, such as embryogenesis and seed settings, have been related more to the levels of Spd than Put (Feng et al., 2011; Chen et al., 2019a). The benefit of Put could be only due to its condition as a precursor of Spd synthesis (Feng et al., 2011). Furthermore, a recent study showed that high endogenous levels of Put but not of Spd could condition grain filling of wheat, and hence, yield, under drought because it induced the accumulation of endogenous ethylene and ABA in the grains, which worsened the adverse stress effects (Liu et al., 2016). Controversial results were also obtained in wheat where the exogenous Spd and Put applications had an opposite effect on florets; while Spd inhibited the floret degeneration, Put enhanced it, resulting in a reduction of fertile floret number (XiaoKang et al., 2016). Another study demonstrated that pretreatment with Spd improved the grain yield of salt-stressed rice plants (Saleethong et al., 2013). However, this response must be concentration-dependent and conditioned by the endogenous levels of the different PA forms and their crosstalk with other phytohormones. For example, the crosstalk between ethylene and PAs has also been reported to condition the seed setting in maize (Feng et al., 2011). These authors also demonstrated that high levels of PAs are needed to avoid aborted kernels. It is well known that ethylene and PAs compete for the same precursor S-adenosyl-methionine (SAM), the methionine-activated form (Podlešáková et al., 2019). This could explain that high PAs and low ethylene levels promote plant flowering and embryogenesis (reviewed by Chen et al., 2019a), where Spd is the leading PA form regulating these processes.

From our knowledge of yield quality in maize, no study focused on the impact of PAs as small molecule-based biostimulants exist in the research literature. Our work demonstrated that PA application improved both yield quantity and quality. The first two parameters analyzed were the CH and protein content (%) in the flour powder obtained from the seeds (Figure 5 and Supplementary Table 6). Regarding CH, it was shown that the crosstalk between PAs and the ethylene pathway could condition plant yield and determine the grain filling and carbohydrate translocation in cereals (Yang et al., 2017). Therefore, we expected PA supplementation to induce a good CH transport, enhancing the kernel set and the final yield. As a result, WD reduced the CH content compared to the WW conditions in untreated plants, as previously observed by Hussain et al. (2020) and Abbas et al. (2021). However, opposite results were also published. For example, it has been reported that drought during the vegetative stage of maize plants induced an increment in glucose and amino acids on the grains (Harrigan et al., 2007) or did not affect the CH content (Barutcular et al., 2016). Interestingly, the PA application increased the flour CH content in the plants under WD conditions over the levels of the WW plants (Figure 5A and Supplementary Table 6). Besides, clear evidence was found about a positive correlation between CH, the leaf RWC, and the Na content, which also positively correlates with the weight of 100 kernels and the total kernels weight (Figure 6B). It can be because the application of PAs under stress has been reported to protect the photosynthetic apparatus (ensuring the synthesis of photosynthates) and increase the osmotic adjustment of the plants under stress conditions (Chen et al., 2019b; Jing et al., 2019). Moreover, Xu et al. (2016) suggested that PA might be involved in the starch biosynthesis in kernels during post-anthesis when the soil is drying. Therefore, it is not surprising that those treatments reported the same yield as the proper irrigated plants and were higher than the WD control plants.

Low water availability increased the protein content in the flour, as described by Lu et al. (2015); Barutcular et al. (2016), and Abbas et al. (2021). However, other studies showed no alterations in the protein content by the water restriction (Hussain et al., 2020). Our work showed that the PA levels could be a relevant factor in determining the protein content in the flour. However, the opposite responses were obtained by the Put or Spd applications under both WW and WD conditions (Figure 5B). There is a direct link between the source–sink ratio during the filling stage and the final protein content in the kernels (Borrás et al., 2002). As mentioned above, Spd is considered essential for a good grain filling. Its exogenous application reduced the production and the flour protein content under WW conditions, whereas it improved the yield but not the protein level under WD (Figures 4, 5B). Contrarily, the Put application enhanced the production and protein content under WW but reduced both under WD. In wheat, the Spd application may affect grain filling by regulating protein synthesis and posttranslational modification, together with a better antioxidative response under drought conditions (Li et al., 2020). This way, the maize–Spd treated plants could deal better with the water limitations, ensuring better production under this adverse condition.

On the other hand, Put exogenous application induced the increment of N content in cotton plants (*Gossypium barbadense* L.) under control conditions and salt stress (Darwish et al., 2013). Our results partially agree since we only reported a protein increment under control conditions. The different behavior observed for Put and Spd applications in the plant growth and yield quantity and quality could be because they regulated the PA synthesis, the conversion, and the terminal catabolism differently. In this regard, it has been proved that at least the synthesis of Spd and Spm *via* the activity of the spermine synthase (SPMS, E.C.2.5.1.22) and spermidine synthase (SPDS, E.C. 2.5.1.16) could condition the flowering (reviewed by Chen et al., 2019b). In addition to the SPDS, the activity of other enzymes related to the PA synthesis, such as ornithine decarboxylase (ODC, E.C. 4.1.1.17) and S-adenosyl-L-methionine decarboxylase (SAMDC, E.C. 4.1.1.50) have also been positively correlated with the grain filling rate (Yang et al., 2020). The PA oxidation *via* polyamine oxidases has been reported to regulate the plant reproductive phases (reviewed by Yu et al., 2019). In this last case, the back conversion from thermospermine or spermine to Spd was the most often identified step conditioning fertility and floral development. Cao et al. (2010) suggested that Spd, among Put and Spm, might be more closely involved in the physiological changes of the maize kernel development. Altogether, it is clear that the effect of the PA application in maize production is due to the endogenous levels and the interconversion between the different PA forms. The further studies are needed focusing on the enzymatic changes to clarify the mode of action of these compounds.

As the last step, we analyzed the mineral content of the flour. Only the limitation of water availability has induced controversial results regarding the flour mineral composition. For example, some studies did not see any effect (Feil et al., 2005), whereas others observed a reduction in K, P, and Fe (Abbas et al., 2021). Contrarily, Avila et al. (2017) reported the increment of P and Mg on grains of maize plants subjected to water limitations. These contrasting results point to a complex response of the plants that affect the metabolism in the grains and condition their mineral composition, which is regulated by the interaction of variety, genotype, and stress intensity. Our study showed that the application with PAs modified the composition under WW and WD (Table 2, Supplementary Table 7, and Figure 7B). Only the Spd treatment increased the Zn content in WW plants, whereas all applications enhanced the Cu under WW and WD. These two minerals have been reported to accumulate under drought stress (da Ge et al., 2010; Avila et al., 2017). Zn deficiency reduces plant growth and nutritional quality (Liu et al., 2019). In a previous experiment with wheat, it was proposed that the increment of N supply also contributed to an enhancement of the Zn concentration in the grains (Kutman et al., 2010).

Copper is also needed for the growth and development of maize and is an essential cofactor for many metalloproteins and various enzymes involved in different physiological and cellular processes such as oxidation and reduction reactions (Rajput et al., 2018). Besides, both minerals are considered essential for human health (White and Broadley, 2009; Garg et al., 2018). It is worth mentioning that the deficiencies in Cu also play a vital role in COVID-19, altering the disease outcomes and prognosis (Altooq et al., 2022). In this context, crop biofortification by applying compounds or fertilizers increases attention to assure plant and human health. Our results showed that a simple PA application could improve the content of these two minerals in the flour, especially when the plants are treated with Spd (five times more Cu under WD or three times more Zn under WW). Additionally, the Spd application also induced a significant accumulation of Ca. However, it is evident that product and crop specificities exist, so it is expected to respond differently to the treatments according to the genotype (Shahrajabian et al., 2021). From the human nutritional point of view, higher Ca content is an advantage because a recent study associates a dietary low calcium intake with a higher risk of all-cause mortality (Yoo et al., 2022). Altogether, we demonstrate that the exogenous application of PAs improves plant performance under stress conditions and can also be an efficient biofortification approach (Figure 7), especially in the case of Spd.

## Conclusion

The polyamine application by drenching can improve maize production under optimal and stress conditions. However, different polyamines induced different responses, conditioned by the growth conditions. Putrescine was the most effective treatment under well-watered conditions because it enhanced the fruit production, the WUE, and the content of Ca and Cu. The spermidine application showed better results under water deficit, with better water balance, water use efficiency, higher production, and better nutritional composition of the flour by increasing carbohydrates content, Cu and Ca. These results point to polyamine supplementation as an exciting approach for crop biofortification. Nevertheless, due to the different effects of genotype and product specificities, a higher effort should be put into elucidating and characterizing the use of these substances for crop and site-specific locations.

## Data availability statement

The original contributions presented in this study are included in the article/Supplementary material, further inquiries can be directed to the corresponding author.

## Author contributions

AH, DJ-A, AB, and NDD designed the idea of the project. AH, DJ-A, and SM-S performed the experiments. AH and NDD analyzed the data and wrote the manuscript. All authors agreed with the last version of the manuscript.
