# Supplementary material for: Addressing the contribution of small molecule-based biostimulants to the biofortification of maize in a water restriction scenario
Source: Front Plant Sci. 2022 Aug 31;13:944066. doi: 10.3389/fpls.2022.944066 (PMC9471082; doi:10.3389/fpls.2022.944066)
Supplement: Supplementary file 1 [file Table_1.PDF]

Supplementary Table S1. Meteorological information obtained from the agrometeorological station placed in the experimental site, for the duration of the experiment. The reported variables are average wind speed (WS; m s^{-1}), maximum wind speed (WS Max; m s^{-1}), average temperature (T; °C), maximum temperature (T Max; °C), minimum temperature (T Min; °C), average relative humidity (RH, %), maximum relative humidity (RH Max; %), minimum relative humidity (RH Min; %), precipitation (P; mm), total daily radiation (Rad; Wh m^{-2}), reference evapotranspiration (ETo; mm). Days marked in red stand for the water deficit period.
(https://www.agrocabildo.org/agrometeorologia_estaciones_detalle.asp?id=3)

Date	WS	WS Max	T Avg	T Max	T Min	RH Avg	RH Max	RH Min	Rain	Solar Rad	Eto
20/05/2021	1.3	3.3	17.4	22.4	11.5	70.3	94.1	32.7	0	8093.2	4.96
21/05/2021	1.5	2.8	19.1	24.2	14.3	58.5	94.1	30.8	0	7903.3	5.24
22/05/2021	1.4	2.5	15.7	18.4	13.5	84.7	95.6	54.8	0	5215.2	3.3
23/05/2021	2.1	3.3	14.2	17.3	11.7	79.8	95.6	60.4	0.7	5899.8	3.41
24/05/2021	2.1	3.3	13.4	16	12.1	85.6	94.9	62.9	0.4	3669	2.48
25/05/2021	2.1	3.7	13.9	17.8	12.5	91.4	96.4	68.5	1.7	3146.5	2.26
26/05/2021	1.7	3.3	13.3	16.3	11.7	87	95	66.1	0.4	3048.8	2.17
27/05/2021	1.9	3.9	14.4	18.6	12.5	81.3	94.2	59.1	0	5892	3.52
28/05/2021	1.6	3.1	14.6	18.5	12.2	76.1	93.3	54.5	0	6309.8	3.73
29/05/2021	1.3	3	14.7	18.6	10.5	80.1	94.5	57.3	0	8382.7	4.48
30/05/2021	1.4	2.8	14.4	16.4	12.9	92.8	96.5	82.9	0.7	2113.3	1.57
31/05/2021	1.1	2.8	14.1	17.7	12.7	89.3	95.7	68.9	1.1	3586.3	2.35
01/06/2021	1.2	3	14.8	18.7	12.1	82	94.1	62	0	5072.2	3.11
02/06/2021	1.3	3.1	15.3	19.4	11.2	78.8	95.6	56	0	5950.5	3.59
03/06/2021	1.3	3.3	15.3	18.9	11.4	85.1	95.6	62.1	1.4	6123.3	3.57
04/06/2021	2.2	4.1	14	17.4	12.2	79.4	93.4	59.9	0	6169.5	3.56
05/06/2021	1.8	3.8	14.1	17.5	10.6	79.4	91.9	55.2	0	6148.7	3.61
06/06/2021	1.4	3.1	14.6	18.5	12.5	82.7	94.2	60.5	0	4895	3.07
07/06/2021	1	4.5	16	20.2	13.6	86.1	95.5	40.5	0	3778.8	2.85
08/06/2021	1.6	5	20.4	29	14.6	63.3	90.7	33.4	0	5844	4.88
09/06/2021	1.8	7.7	22.1	28	18.7	48.5	87.4	27.9	0	3257.5	3.97
10/06/2021	1.5	2.9	23.8	30.9	19.5	42.5	73.2	28.2	0	5478.2	5.15
11/06/2021	2.1	4.2	23.4	28.1	17.4	45.1	67.5	29.7	0	7806.7	6.3
12/06/2021	1.7	3.9	19.6	26	12.8	57.8	95	30.2	0	8493.7	5.75
13/06/2021	1.2	3	15.7	19.6	13.1	84.6	95.6	63.6	0.1	6908.5	3.96
14/06/2021	1.3	3.1	16.2	20	13.5	80	95.6	57.4	0	6699.8	3.99
15/06/2021	1.2	3.2	16.3	21	13.2	79.3	94.9	52.1	0	6790.7	4.12
16/06/2021	1.7	2.9	16.8	19.6	13.5	86	95.7	67.9	0.2	5231.2	3.27
17/06/2021	1.6	3.9	16.4	21	13.7	89.7	95.6	58.1	1.5	3659.8	2.8
18/06/2021	2.2	4.1	15.1	17.1	13.1	87.8	95.6	66.2	3.3	3434.7	2.44
19/06/2021	2.2	3.7	14.2	17.4	12.3	79.4	90	59.9	0	3933.5	2.81
20/06/2021	1.5	3.2	15.1	19.5	12	84.9	95.6	59.2	0.8	6115	3.64
21/06/2021	1.2	2.7	15.4	19.1	13.5	93.7	95.6	77.4	0.6	3163.8	2.16
22/06/2021	1.8	3.3	15.4	19	13.6	82.2	95.6	53.8	0.1	5578.5	3.55

23/06/2021	2.1	3.9	15.4	19	13.1	83.1	95	56	1.7	5833	3.65
24/06/2021	1.8	3.2	14.6	17.8	13	86.5	95	60	0.6	3045.8	2.38
25/06/2021	1.4	2.9	15.5	18.8	13.6	76.3	91.9	59.2	0	6555.7	3.85
26/06/2021	1.1	2.9	15.9	19.4	13.9	81.7	92.6	64.7	0	7290.8	4.15
27/06/2021	1.3	2.9	16	19.7	14	84.8	93.4	67.7	0	5499	3.36
28/06/2021	1	2.8	15.4	19.1	13.5	85.9	94.1	65.3	0	5434.8	3.27
29/06/2021	1.2	2.5	15.7	19	14.4	90.6	96.4	69.9	1.1	4017.8	2.63
30/06/2021	1.1	2.6	15.9	20.1	13.6	88.2	95	67.1	0	5010.5	3.13
01/07/2021	1.1	2.8	16.3	20.2	13.8	80.3	94.9	59.5	0	8556.2	4.78
02/07/2021	1	2.5	16.8	21.3	12.2	80.3	93.5	64.6	0	8369.7	4.73
03/07/2021	1.2	2.3	18.6	25.8	12.1	59.2	94.9	29.9	0	8409.2	5.4
04/07/2021	1.2	2.3	16.7	20.8	11.9	80.6	95.3	36	0.3	6014.8	3.93
05/07/2021	1	2.3	16.7	21.1	13.4	84.3	95.6	63.3	0	7254.8	4.23
06/07/2021	1	2.8	17.1	20.5	15.1	89	95	73.1	0	5444	3.35
07/07/2021	1	2.7	17.7	21.9	14.6	87.8	95	64.8	0.2	6892.3	4.16
08/07/2021	2.1	3.7	16.3	19.6	14.4	89.1	95	65.5	3.1	5095	3.26
09/07/2021	1.9	3.3	15.3	17.7	14	89.1	95	71.2	1	3240.3	2.29
10/07/2021	1.1	2.4	16.2	17.9	14.4	94.1	94.9	89.8	0.7	2357.3	1.67
11/07/2021	0.6	2.1	18.5	24.7	15.1	86.9	95	58.4	0.1	4784	3.26
12/07/2021	3.5	7.2	28.6	33.7	20.2	42.3	79.6	30.6	0	4649	6.37
13/07/2021	2.5	6.6	25.9	33.3	19.1	41	64.5	23.1	0	7103.8	7.2
14/07/2021	1.4	2.9	21.1	25.3	15.8	49.7	74.7	26.5	0	8202.3	5.64
15/07/2021	1.3	2.2	18.5	26.6	13.4	74.3	94.9	22.7	0	8349.3	5.56
16/07/2021	1.2	2.1	20.1	24.3	12.8	53.1	90.5	18.1	0	8116.2	5.3
17/07/2021	1.3	3	22.5	28.1	16.5	49.7	76.3	17.6	0	8017.8	5.82
18/07/2021	4.5	6.3	31.1	35.4	27.3	14.6	17.6	12.3	0	8061.3	11.48
19/07/2021	2.3	4.8	25.3	33.7	12.4	49.4	94.2	13.7	0	8120.8	7.28
20/07/2021	1.1	2.7	17	21.4	14.1	83.5	94.9	43.6	0	7124	4.35
21/07/2021	1.5	3.2	17	21	14.4	81.6	93	61	0	7417.3	4.36
22/07/2021	1.4	3.4	17.5	21.8	13.7	79.2	94.2	55.8	0	8074.3	4.74
23/07/2021	1.3	2.6	17.3	21	15.4	90.3	95.6	71.2	4.1	3534.5	2.51
24/07/2021	1	2.6	17.1	20.4	14.1	90.3	95.6	74.9	0.1	4869.3	3.03
25/07/2021	1.1	2.4	17.9	23.7	12.9	76.4	94.2	44	0	7515	4.65
26/07/2021	1.2	2.5	18.4	23	14.9	84	94.9	57.9	0.5	6660	4.18
27/07/2021	1.6	3.2	16.9	20.5	14.7	89.2	94.9	66.4	1.9	4056.8	2.81
28/07/2021	1.2	2.6	16.8	19.3	15	89.6	94.9	75.6	0.6	3172	2.23
29/07/2021	1	2.3	16.5	19.4	15.3	92	94.9	77.8	0.9	2884.7	2.05
30/07/2021	0.8	2.5	16.9	20.2	15.3	87.7	94.3	70.5	0.1	4032.7	2.66
31/07/2021	1.1	3	17.2	21	15	87.1	94.3	68.3	0	5444.2	3.38
01/08/2021	1.3	2.7	16.3	18.1	15.3	89	94.2	74.2	0	2729.2	2.02
02/08/2021	1.3	2.6	16.6	19.2	14.7	89.5	94.9	72.3	0.6	3147.7	2.25
03/08/2021	1.5	3	17.4	20.6	15.5	86.7	95	61.7	2.4	6317.4	3.85
04/08/2021	1.4	3	17.1	20.6	14.8	82.5	94.1	62.8	0	5143.3	3.3

05/08/2021	1	2.3	17.7	21.7	15.7	91.9	95	72.2	2.4	4497.5	2.93
06/08/2021	1.1	2.8	18.4	21.7	15.5	89.3	95	72.5	3.4	6972.7	4.12
07/08/2021	1.2	2.9	17.8	21.8	15.1	89.5	94.3	66.8	1.1	5194.3	3.33
08/08/2021	1.1	2.6	18.3	21.8	16	84.6	94.3	57.1	2.3	4684.3	3.21
09/08/2021	1.5	3.6	17.1	20.8	14.4	82.8	94.9	57.9	0	6586.7	3.97
10/08/2021	1.2	2.8	17.6	22.5	14	76	94.9	47.2	0	7920.3	4.69
11/08/2021	0.9	2.6	17.2	19.7	15	89.5	94.9	71.1	1	3117.3	2.22
12/08/2021	1.3	2.9	17.8	21.3	15.1	79.2	94.1	61.5	0	5353.5	3.43
13/08/2021	1	2.6	17.9	22.2	14.7	83.7	95	62.5	0	5424.3	3.44
14/08/2021	1.1	2.1	20.6	25.9	15.1	78	95.1	52.5	0	7325.2	4.71
15/08/2021	1.6	4.5	27.7	36.8	19.4	39.2	58.9	18.7	0	6688.8	6.46
16/08/2021	3.7	6.4	32.3	37.6	24.4	21.4	34.4	17.6	0	7208.2	9.54
17/08/2021	5.6	7	31.7	35.9	28.7	15.7	23	10.8	0	7499.2	12.6
