# Supplementary material for: Addressing the contribution of small molecule-based biostimulants to the biofortification of maize in a water restriction scenario
Source: Front Plant Sci. 2022 Aug 31;13:944066. doi: 10.3389/fpls.2022.944066 (PMC9471082; doi:10.3389/fpls.2022.944066)
Supplement: Supplementary file 2 [file Table_2.PDF]

Supplementary Table S2. Biomass (dry weight, mg), relative growth ratio (RGR), and water use efficiency (WUE, mg mL^{-1}) in maize in maize seedlings treated with Put or Spd at five concentrations (0.01, 0.1, 0.5, 1 or 2 mM) grown under optimal conditions (field capacity) or with water limitation (50% field capacity). Mean ± standard error; n stands for the number of seedlings used for the determinations.

Irrigation	Treatment	Biomass	RGR	WUE	n
Field Capacity	Control	0.624 ± 0.016	0.265 ± 0.004	0.011 ± 0.00	24
50% Field Capacity	Control	0.641 ± 0.012	0.269 ± 0.003	0.012 ± 0.00	27
	Put 0.01mM	0.610 ± 0.040	0.257 ± 0.013	0.011 ± 0.001	13
	Put 0.1mM	0.630 ± 0.041	0.262 ± 0.012	0.011 ± 0.001	14
	Put 0.5mM	0.646 ± 0.035	0.268 ± 0.010	0.012 ± 0.001	11
	Put 1mM	0.623 ± 0.031	0.264 ± 0.007	0.011 ± 0.001	13
	Put 2mM	0.615 ± 0.036	0.261 ± 0.009	0.011 ± 0.001	13
	Spd 0.01mM	0.591 ± 0.050	0.249 ± 0.016	0.011 ± 0.001	14
	Spd 0.1mM	0.575 ± 0.058	0.243 ± 0.023	0.010 ± 0.001	10
	Spd 0.5mM	0.605 ± 0.035	0.259 ± 0.009	0.011 ± 0.001	13
	Spd 1mM	0.589 ± 0.038	0.252 ± 0.012	0.010 ± 0.001	15
	Spd 2mM	0.587 ± 0.046	0.249 ± 0.015	0.010 ± 0.001	14
