# Supplementary material for: Addressing the contribution of small molecule-based biostimulants to the biofortification of maize in a water restriction scenario
Source: Front Plant Sci. 2022 Aug 31;13:944066. doi: 10.3389/fpls.2022.944066 (PMC9471082; doi:10.3389/fpls.2022.944066)
Supplement: Supplementary file 3 [file Table_3.PDF]

Supplementary Table S3. RWC variation (%) in maize plants treated with 0.1 mM Put or 0.5 mM Spd under optimal conditions (WW) or water deficit (WD) at t_1 (15 days after the stress onset) and t_2 (30 days after the stress onset). Positive and negative values mean increase and decrease, respectively.

		RWC Variation	
		t_1	t_2
WW	Put	4%	-0.46%
	Spd	2.60%	1.55%
WD	Put	8.71%	14.90%
	Spd	4.14%	15.81%
