# Supplementary material for: Addressing the contribution of small molecule-based biostimulants to the biofortification of maize in a water restriction scenario
Source: Front Plant Sci. 2022 Aug 31;13:944066. doi: 10.3389/fpls.2022.944066 (PMC9471082; doi:10.3389/fpls.2022.944066)
Supplement: Supplementary file 4 [file Table_4.PDF]

Supplementary Table S4. Biomass parameters [stem diameter (mm), plant length (cm), the flag leaf length (cm) and width (cm), and the ratio length/ width for the flag leaf (L/W)] in maize plants untreated (Control) or treated with 0.1 mM Put or 0.5 mM Spd grown under optimal conditions (WW) or water deficit (WD). Mean ± standard error (s.e.); n stands for the number of seedlings used for the determinations. Different letters indicate significant differences between the treatments and growth conditions according to the LSD test after two-way ANOVA , $p<0.05$.

		Stem Diameter		Plant Length		Flag Leaf Length		Flag Leaf Width		Flag Leaf L/W	
		mean ± s.e.	n	mean ± s.e.	n	mean ± s.e.	n	mean ± s.e.	n	mean ± s.e.	n
WW	Control	19.6 ± 0.8 **b**	6	289.2 ± 19.0 **ab**	6	77.6 ± 5.7 **bc**	12	7.8 ± 0.3 **ab**	12	9.8 ± 0.8 **b**	12
	Put	16.8 ± 0.9 **ab**	8	312.8 ± 7.2 **b**	9	80.4 ± 3.2 **c**	15	7.9 ± 0.3 **b**	15	10.3 ± 1.0 **b**	15
	Spd	18.2 ± 1.2 **ab**	6	290.5 ± 13.7 **ab**	6	74.4 ± 4.0 **bc**	10	7.3 ± 0.3 **ab**	10	10.2 ± 1.5 **b**	10
WD	Control	18.2 ± 1.2 **ab**	6	286.2 ± 8.9 **ab**	6	66.4 ± 5.0 **ab**	12	6.9 ± 0.4 **a**	12	9.6 ± 0.4 **b**	12
	Put	16.0 ± 1.4 **a**	5	280.8 ± 15.1 **ab**	6	76.8 ± 3.6 **bc**	10	7.9 ± 0.4 **ab**	10	9.8 ± 0.3 **b**	10
	Spd	18.3 ± 0.7 **ab**	7	271.7 ± 10.3 **a**	7	54.4 ± 8.8 **a**	7	7.0 ± 0.6 **ab**	7	7.5 ± 0.6 **a**	7
