# Supplementary material for: Addressing the contribution of small molecule-based biostimulants to the biofortification of maize in a water restriction scenario
Source: Front Plant Sci. 2022 Aug 31;13:944066. doi: 10.3389/fpls.2022.944066 (PMC9471082; doi:10.3389/fpls.2022.944066)
Supplement: Supplementary file 5 [file Table_5.PDF]

Supplementary Table S5. Production related [cobs per plant (Cobs/Pl), average of the cobs fresh weight (Cob FW, g), average of the cob dry weight (Cob DW,g), number of kernels along the length of the cob (L), number of kernels along the circumference of the cob (Circ.), cob diameter (Diam., mm), average weight of 100 dry kernels (100 k DW, g), average weight of the total number of kernels per cob (Total KW, g), average of the total number of kernels per cob (TK), yield per hectare considering the kernel weight (Yield, g (x10^6) ha^{-1}), harvest index (HI), and production water use efficiency ($WUE_{p,}$)] in maize plants untreated (Control) or treated with 0.1 mM Put or 0.5 mM Spd grown under optimal conditions (WW) or water deficit (WD). Mean ± standard error (s.e.); n stands for the number of seedlings used for the determinations. Different letters indicate significant differences between the treatments and growth conditions according to the LSD test after two-way ANOVA , $p<0.05$.

		Cobs/Pl		Cob FW		Cob DW		L		Circ.		Diam.	
		mean ± s.e.	n	mean ± s.e.	n	mean ± s.e.	n	mean ± s.e.	n	mean ± s.e.	n	mean ± s.e.	n
WW	Control	1.22 ± 0.1 **bc**	65	135.3 ± 5.4 **a**	29	59.2 ± 3.3 **a**	7	37.7 ± 1.9 **c**	7	12.6 ± 0.4 **a**	7	27.5 ± 0.6 **a**	7
	Put	1.26 ± 0.1 **c**	77	159.5 ± 7.1 **bc**	20	58.9 ± 4.0 **a**	8	29.0 ± 0.7 **a**	8	13.0 ± 0.4 **ab**	8	28.2 ± 0.8 **a**	8
	Spd	1.18 ± 0.1 **bc**	50	168.7 ± 5.3 **bcd**	35	57.3 ± 3.2 **a**	7	30.1 ± 1.2 **ab**	7	12.6 ± 0.4 **a**	7	28.1 ± 1.0 **a**	7
WD	Control	0.88 ± 0.1 **a**	40	176.6 ± 5.9 **cd**	30	54.1 ± 5.0 **a**	7	34.1 ± 1.6 **bc**	7	14.4 ± 0.5 **c**	7	28.5 ± 0.8 **a**	7
	Put	1.11 ± 0.1 **bc**	28	154.9 ± 7.2 **b**	26	56.9 ± 3.4 **a**	6	28.2 ± 2.2 **a**	6	13.3 ± 1.0 **bc**	6	29.2 ± 0.7 **a**	6
	Spd	1.1 ± 0.0 **b**	52	193.0 ± 12.1 **d**	8	60.5 ± 5.0 **a**	6	35.2 ± 1.4 **c**	6	13.3 ± 0.6 **bc**	6	27.9 ± 0.9 **a**	6

		100 K DW		Total KW		TK		Yield		HI		WUE_p	
		mean ± s.e.	n	mean ± s.e.	n	mean ± s.e.	n	mean ± s.e.	n	mean ± s.e.	n	mean ± s.e.	n
WW	Control	10.3 ± 0.1 **b**	18	37.9 ± 2.3 **a**	6	368.5 ± 21.8 **a**	6	1.6 ± 0.3 **b**	6	0.24 ± 0.1 **c**	6	0.55 ± 0.0 **a**	29
	Put	10.5 ± 0.3 **b**	24	36.1 ± 2.3 **a**	8	342.9 ± 21.9 **a**	8	1.8 ± 0.1 **b**	8	0.23 ± 0.0 **bc**	8	0.67 ± 0.0 **b**	20
	Spd	10.3 ± 0.5 **b**	21	34.1 ± 2.9 **a**	7	332.1 ± 28.5 **a**	7	1.6 ± 0.1 **b**	7	0.2 ± 0.0 **ab**	7	0.66 ± 0.0 **b**	35
WD	Control	9.0 ± 0.5 **a**	21	29.2 ± 3.3 **a**	7	326.0 ± 36.7 **a**	7	1.0 ± 0.1 **a**	7	0.17 ± 0.0 **a**	7	0.52 ± 0.0 **a**	30
	Put	10.2 ± 0.2 **b**	18	34.8 ± 2.7 **a**	6	339.8 ± 26.0 **a**	6	1.5 ± 0.1 **b**	6	0.22 ± 0.0 **bc**	6	0.57 ± 0.0 **a**	26
	Spd	10.0 ± 0.3 **b**	18	36.4 ± 4.6 **a**	6	364.4 ± 46.0 **a**	6	1.6 ± 0.2 **b**	6	0.19 ± 0.0 **ab**	6	0.71 ± 0.04 **b**	8
