# Supplementary material for: Addressing the contribution of small molecule-based biostimulants to the biofortification of maize in a water restriction scenario
Source: Front Plant Sci. 2022 Aug 31;13:944066. doi: 10.3389/fpls.2022.944066 (PMC9471082; doi:10.3389/fpls.2022.944066)
Supplement: Supplementary file 6 [file Table_6.PDF]

Supplementary Table S6. Content of carbohydrates and proteins (%) in maize plants untreated (Control) or treated with 0.1 mM Put or 0.5 mM Spd grown under optimal conditions (WW) or water deficit (WD). Mean ± standard error (s.e.); n stands for the number of seedlings used for the determinations. Different letters indicate significant differences between the treatments and growth conditions according to the LSD test after two-way ANOVA , $p<0.05$.

		Carbohydrates		Proteins	
		mean ± s.e.	n	mean ± s.e.	n
WW	Control	23.8 ± 0.8 **bc**	12	14.6 ± 0.5 **b**	3
	Put	23.1 ± 1.0 **ab**	12	18.1 ± 0.4 **d**	3
	Spd	23.1 ± 1.0 **ab**	12	13.3 ± 0.5 **a**	3
WD	Control	20.7 ± 0.7 **a**	12	16.8 ± 0.1 **c**	3
	Put	26.5 ± 1.3 **c**	11	14.2 ± 0.3 **ab**	3
	Spd	25.6 ± 1.0 **bc**	12	16.8 ± 0.4 **c**	3
