# Supplementary material for: Addressing the contribution of small molecule-based biostimulants to the biofortification of maize in a water restriction scenario
Source: Front Plant Sci. 2022 Aug 31;13:944066. doi: 10.3389/fpls.2022.944066 (PMC9471082; doi:10.3389/fpls.2022.944066)
Supplement: Supplementary file 7 [file Table_7.PDF]

Supplementary Table S7. Mineral content (ppm) in maize plants untreated (Control) or treated with 0.1 mM Put or 0.5 mM Spd grown under optimal conditions (WW) or water deficit (WD). Mean ± standard error (s.e.); n stands for the number of seedlings used for the determinations. Different letters indicate significant differences between the treatments and growth conditions according to the LSD test after two-way ANOVA , $p<0.05$.

		Ca		Fe		K		Mg	
		mean ± s.e.	n	mean ± s.e.	n	mean ± s.e.	n	mean ± s.e.	n
WW	Control	75.6 ± 17.0 **ab**	4	37.9 ± 25.0 **a**	8	4239.5 ± 379.2 **ab**	8	864.0 ± 57.5 **ab**	8
	Put	113.3 ± 39.7 **ab**	3	9.4 ± 1.2 **a**	4	3166.9 ± 262.9 **a**	4	730.7 ± 69.8 **a**	4
	Spd	86.7 ±3.8 **ab**	4	49.2 ± 38.0 **a**	4	3516.4 ± 120.4 **ab**	4	799.4 ± 44.4 **a**	4
WD	Control	56.6 ± 13.8 **a**	5	23.2 ± 7.4 **a**	7	5316.3 ± 364.9 **c**	7	1026.6 ± 46.1 **c**	7
	Put	68.7 ± 36.0 **ab**	3	42.6 ± 19.7 **a**	4	4676.5 ± 477.4 **bc**	4	1004.6 ± 68.5 **bc**	4
	Spd	124.3 ± 0.8 **b**	4	10.4 ± 3.8 **a**	4	3776.3 ± 263.4 **ab**	4	778.5 ± 30.3 **a**	4

		Na		P		Zn		Cu	
		mean ± s.e.	n	mean ± s.e.	n	mean ± s.e.	n	mean ± s.e.	n
WW	Control	332.5 ± 12.1 **b**	8	1396.4 ± 43.1 **ab**	8	26.7 ± 7.5 **a**	8	3.9 ± 0.7 **a**	8
	Put	332.9 ± 5.6 **b**	4	1261.6 ± 44.1 a	4	7.8 ± 1.1 **a**	4	6.7 ± 1.2 a	4
	Spd	316.8 ± 6.2 **b**	4	1536.8 ± 19.9 **bc**	4	73.0 ± 46.7 **b**	4	4.9 ± 0.3 **a**	4
WD	Control	286.5 ± 2.7 **a**	7	1646.0 ± 72.8 **c**	7	20.1 ± 3.9 **a**	7	9.2 ± 2.0 **a**	7
	Put	340.5 ± 10.4 **b**	4	1640.7 ± 105.1 **c**	4	19.1 ± 2.7 **a**	4	4.5 ± 0.5 **a**	4
	Spd	326.2 ± 8.7 **b**	4	1316.6 ± 70.7 **a**	4	9.3 ± 1.8 **a**	4	21.5 ± 9.6 **b**	4
